# Deep Neural Network Probabilistic Decoder for Stabilizer Codes

**DOI:** 10.1038/s41598-017-11266-1

**Published:** 2017-09-08

**Authors:** Stefan Krastanov, Liang Jiang

**Affiliations:** 1 Departments of Physics and Applied Physics, Yale University, New Haven, 06520 Connecticut USA; 2 Yale Quantum Institute, Yale University, New Haven, 06520 Connecticut USA

## Abstract

Neural networks can efficiently encode the probability distribution of errors in an error correcting code. Moreover, these distributions can be conditioned on the syndromes of the corresponding errors. This paves a path forward for a decoder that employs a neural network to calculate the conditional distribution, then sample from the distribution - the sample will be the predicted error for the given syndrome. We present an implementation of such an algorithm that can be applied to any stabilizer code. Testing it on the toric code, it has higher threshold than a number of known decoders thanks to naturally finding the most probable error and accounting for correlations between errors.

## Introduction

Constructing a physical computing machine, whether a classical or a quantum one, requires, inescapably, the implementation of an error correcting mechanism that guards against the noise picked up from the environment and the imperfections in the operations being performed^1–3^. Early in the development of both classical and quantum computers, “threshold” theorems were proven to show the existence of encoding schemes which reliably store and process information in a “logical” set of bits (or qubits), by encoding it redundantly on top of a bigger set of less reliable “physical” bits (or qubits), as long as the error rate on the physical layer is smaller than a fixed threshold^4, 5^. The vast majority of quantum error correcting codes fall in the class of stabilizer codes (a generalization of the classical linear codes)^6^. They are characterized by the group of stabilizer operators that preserve the logical states (similarly to the list of constraints represented by the parity check matrix H for classical linear codes). The list of nontrivial stabilizer operator measurements (or violated parity constraints for a classical code) is called the syndrome of the error. While providing for efficient encoding, linear and stabilizer codes do not necessarily have known efficient decoding algorithms that can deduce from a given syndrome what errors have occurred.

In the general case decoding a stabilizer code is an NP-hard problem. An active area of research is the design of codes with some additional algebraic structure that permits efficient decoders, but still retains high rates (ratio of logical to physical qubits) with acceptable distances (maximal number of correctable errors on the physical qubits). Schemes like the CSS approach^7–9^ permit the creation of quantum codes from classical codes, but they do not guarantee that the decoder that worked for the classical code still works for the quantum one. A particularly interesting example is the class of LDPC codes^10, 11^ which are high-performing classical codes with efficient decoders, however those decoders do not work for the quantum LDPC codes^12^.

**Figure 1:**
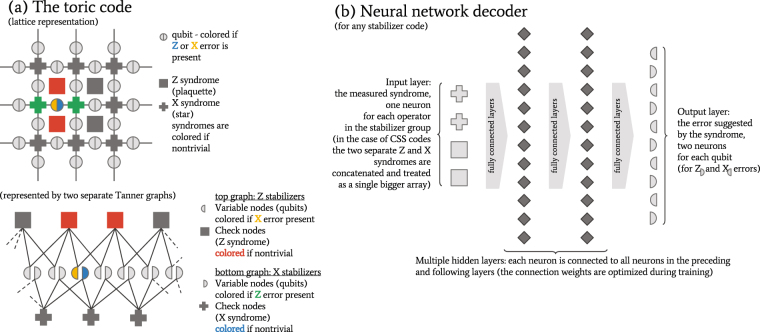
Quantum Error Correcting Codes. A very general class of QEC codes is the class stabilizer codes, defined by the stabilizer subgroup of the physical qubits that leaves the state of the logical qubits unperturbed. Our neural architecture can be readily applied to such codes, however many codes of practical interest (like the one we are testing against) have additional structure that would be interesting to consider. The example in (**a**) shows a small patch of a toric code, which is a CSS code (the stabilizer operators are products of only Z or only X operators, permitting us to talk of Z and X syndromes separately). Moreover, the toric code possesses a lattice structure that provides for a variety of decoders designed to exploit that structure. Our decoder, depicted in (**b**), does not have built-in knowledge of that structure, rather it learns it through training. Due to size constraints, the depictions present only a small subset of all qubit or syndrome nodes.

Here we present a decoding algorithm that can be applied to any stabilizer code — the decoder employs machine learning techniques to “learn” any structures that would make the approximate decoding problem easier than the general NP-hard decoding problem: it “learns” the probability distributions of errors conditioned on a given syndrome and efficiently uses samples from that distribution in order to predict probable errors. The conditional probability distribution is encoded in a deep neural network. The “learning” involves training the neural network on pairs of errors and corresponding syndromes (generated from an error model for the physical qubits and a parity check matrix for the code in use). We test the algorithm on the toric code (Fig. 1a) definied on a two-dimensional lattice on a torus^13^. Since the toric code has low-weight local stabilizers, it is also a quantum LDPC code with structure that impedes typical belief propagation algorithms. Our decoder significantly outperforms the standard “minimal-weight perfect matching” (MWPM) decoder^14, 15^. Moreover, it has comparable threshold with the best renormalization group decoders^16^. For code-sizes up to 200 physical qubits the decoder is practical and we discuss how to extend our neural network architecture to negate the inefficiencies that kick in at that stage.

Machine learning techniques, specifically neural networks, have been gaining popularity over the last year, in particular with the recent developments in using restricted Boltzmann machines for describing the ground state of many-body systems^17^ or convolutional networks for identifying phases of matter^18^. A preprint on the use of restricted Boltzmann machines to decoding the toric code has been available for a few months as well^19^, however that architecture does not yet outperform known decoders like MWPM and has been tested only on the Z syndrome on lattices no bigger than 5-by-5. At the time of submission of this manuscript two other related preprints were made available: a fast neural network decoder for small surface codes, that however also does not significantly outperform MWPM^20^, and a recurrent neural network decoder outperforming MWPM as evaluated on a 17 qubit surface code^21^. It is worth noting as well that over the last few months work has started on deep learning methods for decoding classical algebraic codes^22^.

## Results

**Figure 2:**
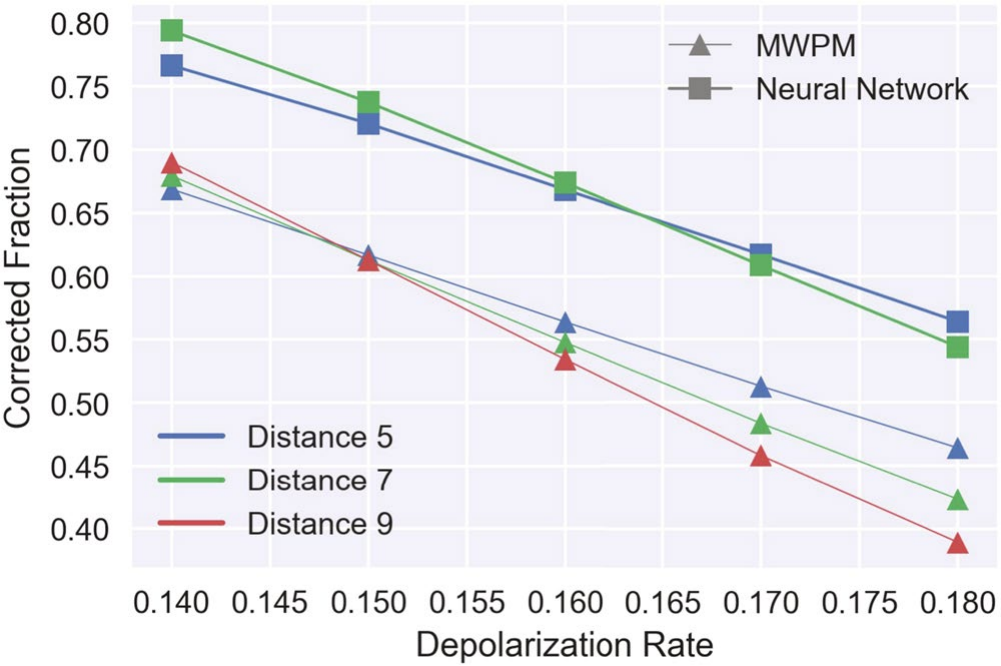
Decoder performance for toric codes of distances 5 and 7. The x axis is the depolarization rate of the physical qubits (the probability that an X, Y, or Z error has occurred), while the y axis is the fraction of properly decoded code iterations (the conjugate of the logical error rate). The neural network decoder (rectangular markers) significantly outperforms the minimal weight perfect matching decoder (triangular markers), both in terms of threshold and logical error rate. For the above plots, neural networks with 18 hidden layers were used.

For testing purposes we trained our neural decoder (depicted in Fig. 1b) on the toric code, which already has a number of known decoding algorithms specifically tuned for its lattice structure. The evaluation was done under the depolarization error model. Our algorithm significantly outperforms the standard MWPM decoder. The comparison of the two decoders in Fig. 2 shows a threshold single-qubit error which is nearly 2 percentage points higher for the new algorithm (around 16.4% for the depolarization error model), and the fraction of correctly decoded errors is consistently around 10 percentage points higher than the fraction of errors corrected by MWPM. Furthermore, the neural decoder threshold compares favorably to renormalization group decoders^16^ (threshold of 15.2%), and decoders explicitly tuned to correlations between Z and X errors^23^ (threshold of 13.3% for a triangular lattice, as it is tuned for asymmetric codes). To our knowledge only a renormalization group decoder^24^ enhanced by a sparse code decoder^12^ reaches a similar threshold (16.4%). It is worth noting that the sampling procedure in our decoder makes it impractically slow for codes of more than 200 physical qubits, while other decoders remain practical. On the other hand, the neural architecture is versatile enough to be applied to any stabilizer code, unlike the other decoders discussed here, which are limited to only topological codes. The best of both worlds — record threshold and fast decoding — should be achievable if we couple the renormalization decoder of^24^ with our neural decoder (instead of the currently suggested sparse code decoder^12^), however this will be applicable only to topological codes. We discuss other ways to avoid the inefficiencies in our decoder without compromising its ability to “learn” to decode any stabilizer code.

After being properly trained for a given error rate of a particular error model, the neural network at the heart of our decoder becomes a compact approximate representation of the probability distribution of errors that can occur. The decoding algorithm consist of inputing the measured syndrome in the neural network, interpreting the output as a probability distribution of the errors conditioned on the given syndrome, and repeatedly sampling from that distribution. The performance of the decoder scales monotonically with the size of the network, up to a point of diminishing returns where using more than about 15 hidden layers (for a distance 5 code) stops providing improvements.

**Figure 3:**
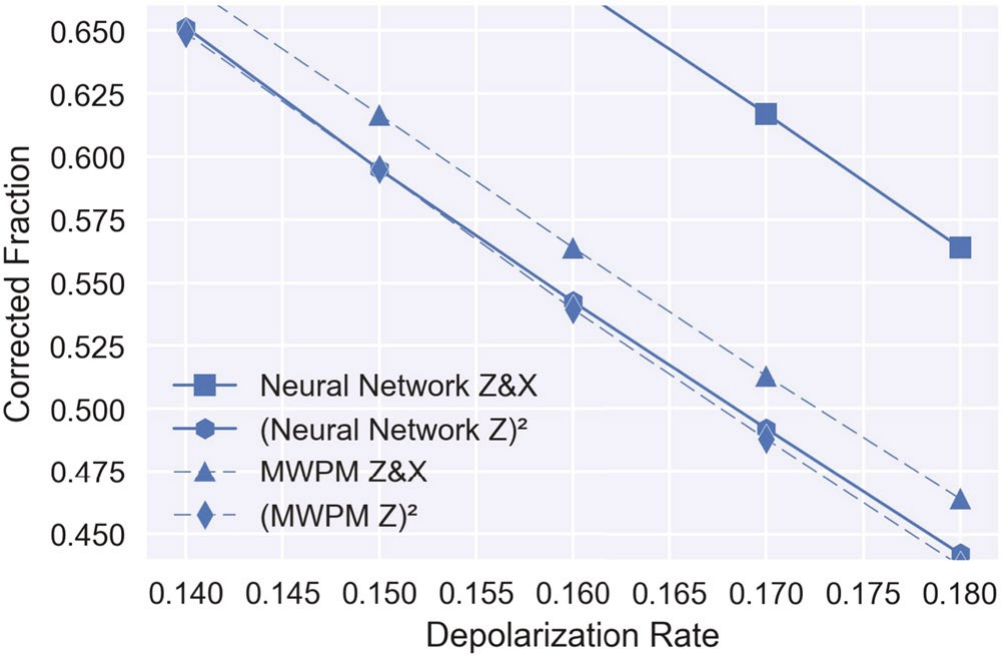
Correlations learned by the neural network. The neural network and MWPM decoder performances for a distance 5 code from Fig. 2 are repeated in this plot. To visualize the importance of taking into account correlations between errors, we also plot the square of the “corrected fraction” for a neural and a MWPM decoder decoding only the Z stabilizer (this neural decoder was trained only on Z stabilizer data). The neural decoders outperforms MWPM both when decoding only the Z stabilizer and when decoding Z and X together. If there were no correlations between errors then the squared value for decoding Z would be the same as the value for decoding both Z and X for each decoder. However, the difference is much more substantial between the two neural decoders, demonstrating the limitations of MWPM and similar decoders that do not account for correlations.

The significant gain in the threshold value relative to some known decoders can be traced to two characteristics of the neural network (discussed in more details in the Methods section). Firstly, the neural network is trained on (stabilizer, error) pairs generated from the error model, therefore it is optimized directly for producing “most probable error”, not for finding an imperfect proxy like “error with lowest energy” as is the case for MWPM. Secondly (depicted in Fig. 3), it learns the Z and X stabilizers together, hence it can encode correlations between them in its structure. Namely, in a typical depolarization error models, one third of the errors are Y errors (equivalent to both X and Z error happening), therefore the knowledge of this correlation can be a useful resource for decoding. Other decoders need significant modifications to even partially employ those correlations in decoding^23^.

## Methods

Neural networks are particularly efficient tools for function approximation^25^, where a function *f:x→f*(*x*) is to be learned from large amount of training data given in the form of pairs (*x*,*f*(*x*)). The input *x* is set as the value of the input layer of neurons. Each of those neurons is connected through axons with each neuron of the next layer (the first “hidden” layer). Multiple hidden layers of neurons can be connected together in this fashion in order to construct a deeper neural network. The last layer of the network is the output layer - its value represents *f*_*learned*_(*x*). The value of a neuron (i.e. its activation value) is calculated as a weighted sum of the activation values of the neurons connected to it from the previous layer. That sum is then passed through a non-linear function (called the activation function). This activation value is then further passed on to the neurons of the next layer, where the process is repeated until it reaches the output layer. The weights in the sums (i.e. the strength of connections between the neurons) are parameters which are optimized through stochastic gradient descent in order to minimize the distance between *f*_*learned*_ and *f* calculated on the training data. The choice of activation function, the size of the hidden layers, and the step size for gradient descent (also called the hyperparameters) are decided in advance, before training. Current best practices include performing a random search to find the best hyperparameters.

In the particular case of decoding a stabilizer quantum error correcting code we want to map syndromes to corresponding physical errors, hence, we take the input layer to be the syndrome (obtained from measuring the stabilizers). For instance, for a toric code of lattice size 9-by-9 we have to measure 81 plaquette operators and 81 star operators for a total of 162 input neurons (having value 0 if the syndrome is trivial and 1 if not). Similarly, we set the output layer to be the prediction for what physical errors occurred (typically represented in the Heisenberg picture, thanks to the Gottesman– Knill theorem). Using the same example, we have 162 physical qubits and we need to track their eigenvalues under both Z and X operators, requiring a total of 324 output neurons (having value 0 if no error has occurred and value 1 otherwise).

To completely define the neural network architecture we set the activation functions of the hidden layers to tanh and the activation of the output layer to the sigmoid function *σ*(*x*) = (1 − *e* ^−*x*^)^−1^∈[0, 1]. The size of the hidden layer was set to four times the size of the input layer. These decisions were reached after an exhaustive search over possible hyperparameters tested on toric codes of distance 3 to 6, and proved to work well for bigger codes as well. The number of hidden layers was varied - deeper networks produce better approximations up to a point of diminishing returns around 15 layers. The step size for the gradient descent (a.k.a. the learning rate) was annealed - gradually lowered, in order to permit rapidly reaching the minimum. The distance measure between training and evaluation data that is being minimized by the gradient descent is their binary crossentropy (a measure of difference between two probability distributions discussed below).

The training was done over one billion (syndrome, error) pairs in batches of 512, taking about a day of GPU wall time for a 5-by-5 toric code. The pairs were generating on the fly, by first generating a sample error from the given error model (this training set can also be efficiently generated directly on the experimental hardware), and then obtaining the corresponding syndrome by a dot product with the parity check matrix. The error model used for each physical qubit was qubit depolarization, parametrized by qubit fidelity *p* - the probability of no error happening on a given qubit, or equivalently depolarization rate 1 − *p*. Under this model, Z, X, and Y (consecutive Z and X) errors had equal probabilities of ^1^/_3_(1 − *p*). For each value of p we trained a new network, however the results showed some robustness to testing a neural network at an error rate different from the one at which it was trained.

The performance of the network was improved if we normalize the input values to have an average of 0 and a standard deviation of 1. For a depolarization error rate 1 − *p*, the rate at which a Z eigenvalue flips is *P*_e_ = ^2^/_3_(1 − *p*) and independently the rate for X flips is the same. In the example of the toric code the rate of non-trivial stabilizer measurements will be the same for Z and for X, namely *P*_*s*_ = 4*q* ^3^(1 − *q*) + 4*q*(1 − *q*)^3^ and the variance will be *V*_*s*_ = *P*_*s*_ − *P*_*s*_^2^.

**Figure 4:**
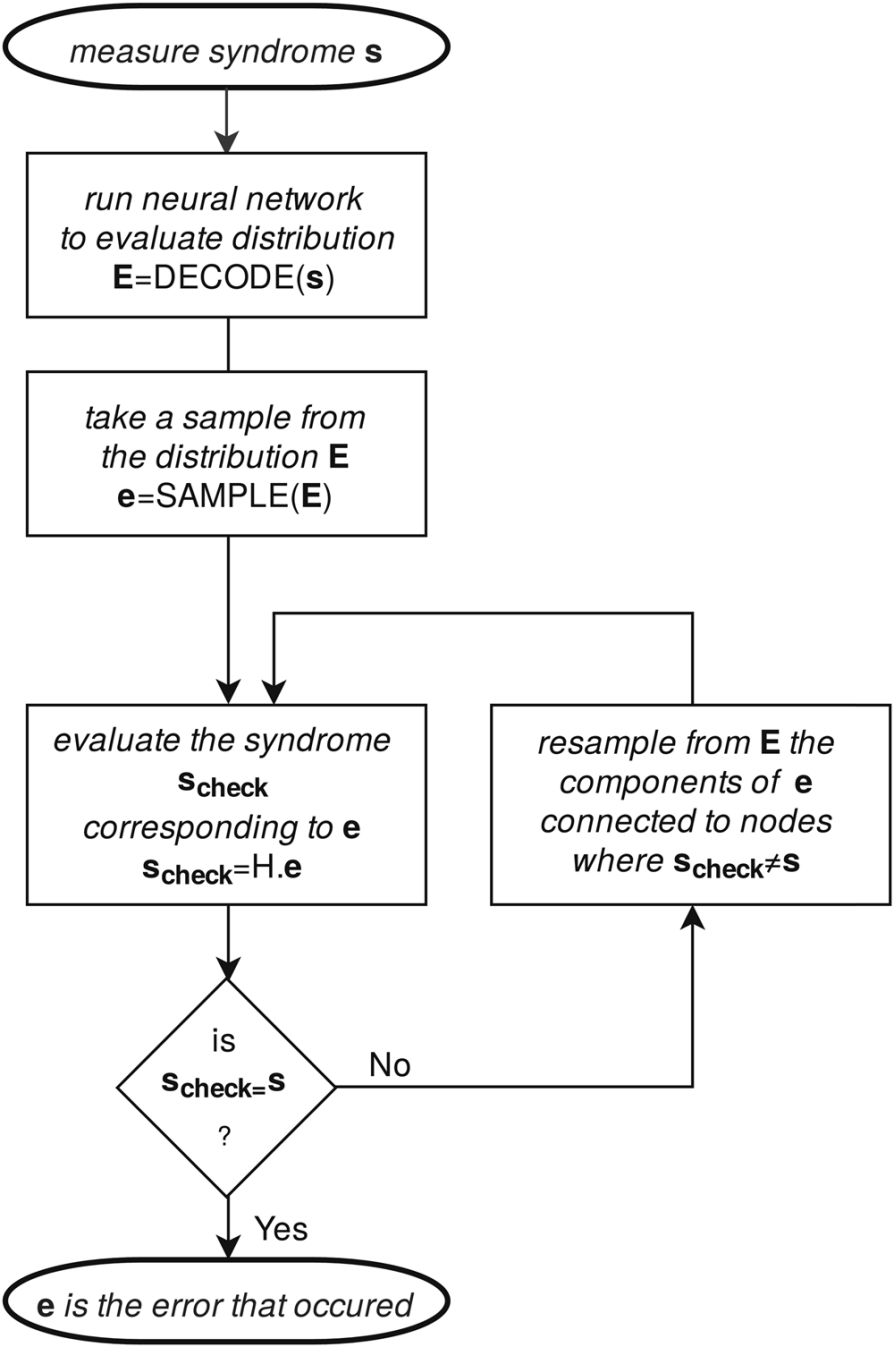
Sampling the neural network. (Arrays in the diagram are in bold font, *H* is the parity check matrix of the code) After the neural network is trained its output can efficiently be evaluated for any given syndrome *s*. The output array *E* is interpreted as a list of probabilities for each qubit for an error to have happened. An array *e* (whether an error occurred at each qubit) is sampled from *E*. In the loop we check whether the guess *e* actually produces the same syndrome as the initially given one. If not, we resample only the qubits taking part in the stabilizer measurement corresponding to the incorrect elements of the syndrome. If the loop runs for more than a set number of iterations we declare failure to decode (a detected, however not corrected, error). As for any other decoding algorithm the final result may be wrong if the total error that occurred is of particularly low probability (i.e. of high weight). In the case of a general stabilizer code *H* stands for the list of stabilizer operators.

At this point we have not discussed yet how to use the fully trained neural network in decoding. A trained network can efficiently evaluate the approximation of the decoding function (from here on referred to as DECODE: syndrome → error), so all Alice needs to do in order to perform error correction on her quantum memory is to measure the syndrome and run the neural network forward to evaluate DECODE(syndrome). However, the neural network is a continuous function and an imperfect approximation, therefore the values in DECODE(syndrome) will not be discrete zeros and ones, rather they will be real numbers between zero and one. A common way to use and interpret those values is to view them as a probability distribution over possible errors, i.e. the i-th value in the array DECODE(syndrome) is a real number between zero and one equal to the probability of the i-th qubit experiencing a flip (half of the array corresponds to Z errors and half of the array corresponds to X errors). This interpretation is reinforced by our use of binary crossentropy as an optimization target during training. In order to deduce what error has occurred we sample this probability distribution. We verify the correctness of the sample by computing the syndrome that the predicted error would cause - if it differs from the given syndrome we resample. This sampling procedure is present in ref. 19 as well, however we further employ a simple “hard decision belief propagation/message passing” sampling, which can speed up the sampling process by an order of magnitude: we resample only the qubits taking part in the stabilizer measurement corresponding to the incorrect elements of the syndrome (Fig. 4).

### Data availability

The code for building, training, and evaluating the neural network decoder is publicly available on the authors’ web page, and shell scripts with the parameters for the presented figures are available upon request. Pretrained neural networks can be provided as well.

## Discussion

On first sight our decoder implementation can look like a look-up table implementation, however we would like to stress the immense compression of data that the neural network achieves. Firstly, one can consider the size of the neural network itself. For a code on *N* physical qubits the number of parameters needed to describe a neural decoder of *L* layers will be ${\mathscr{O}}({N}^{2}L)$ or on the order of thousands for the codes we tested. Moreover, the size of the training dataset for the codes we tested did not exceed 10 billion, and it can be made orders of magnitude smaller if we reuse samples in the stochastic gradient descent (a common approach taken in training). On the other hand, the size of a complete lookup table would be on the order of ${\mathscr{O}}({4}^{N})$. Even if we take only the most probable errors (and discard the errors that have less than 5% chance of occurring), at depolarization rate of 0.1 we need a lookup table bigger than 10^12^ for a distance 5 toric code (50 qubits), bigger than 10^23^ for distance 7 toric code (98 qubits), and bigger than 10^37^ for distance 9 toric code (162 qubits).

Thanks to this compression, to the direct optimization for most probable error, and to the ease of including knowledge of error correlations in the decoding procedure, the algorithm presented here is one of the best choices for decoding stabilizer codes of less than 200 qubits. While we used the toric code for our testing, there is nothing in our design that has knowledge of the specific structure of that code - the neural decoder can be applied to the decoding of any stabilizer code.

**Figure 5:**
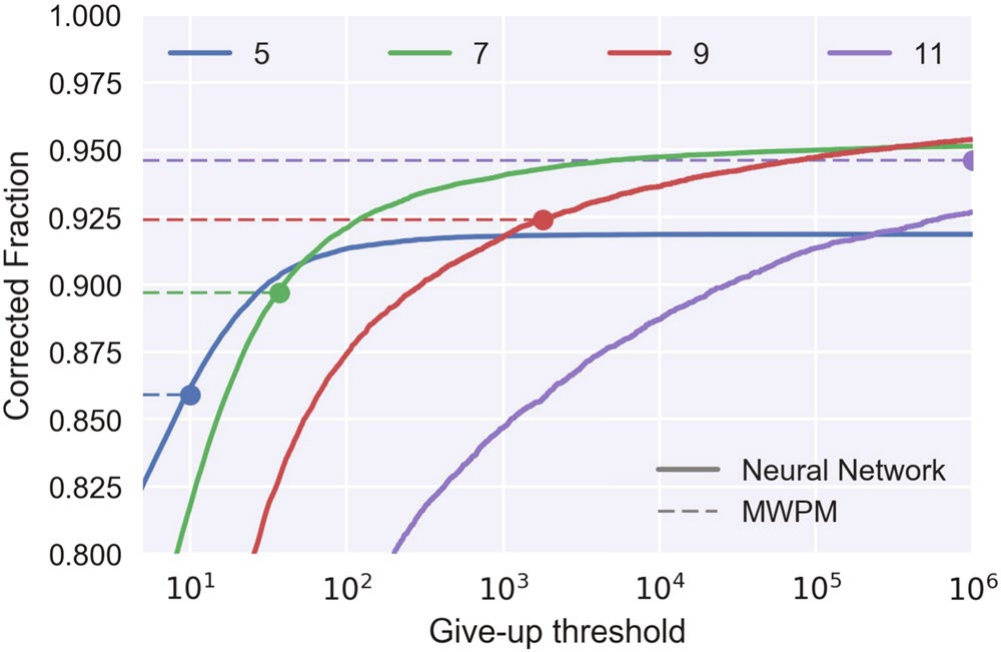
Sampling overhead versus decoder performance. Sampling possible errors from the output of the neural network is an iterative process not guaranteed to reach an acceptable solution, therefore we need to set an upper bound on how many iterations are permitted before giving up (which would result in a detected but not corrected error). The plot gives the performance of our decoder trained on toric codes of different distances with respect to the maximal permitted number of iterations. The dashed lines give the MWPM decoder performances for the same codes as a reference. Codes up to distance 9 (containing 162 physical qubits) are practical, but extending using our decoder for codes with more than 242 physical qubits would be prohibitive due to the sampling overhead. The evaluations were done for 10% depolarization rate on the physical qubits.

Due to the probabilistic nature of the sampling, the decoder becomes impractically inefficient for codes bigger than roughly 200 qubits as one can see in Fig. 5. This can be attributed to two characteristics of our algorithm: we use a simple hard-decision message passing algorithm in our sampling instead of a more advanced belief propagation algorithm seeded by output of the neural network; additionally, our neural network learns only the marginal probabilities for errors on each qubit, without providing the correlations between those errors. A more advanced neural network could address this problem by providing correlation information in its output layer. Our focus forward goes beyond that: we can consider recurrent generative networks^26^ that have the belief propagation as part of their recurrent structure.

While this decoder is general and it can be applied to any stabilizer code, one can also design neural network architectures that specifically exploit the lattice structure and translational symmetry of the toric code. For instance, convolutional neural networks are well adapted for processing 2D data. Moreover thanks to the translational symmetry one can envision a decoder that is trained on a fixed patch of the code and it can be used for toric codes of any size. As already mentioned, our decoder can readily replace the sparse code decoder^12^ used as part of the renormalization group decoder of ^24^, hence providing great decoding speed and high threshold values.

## Electronic supplementary material

Supplementary Material
