# Supplementary material for: Deep Neural Network Probabilistic Decoder for Stabilizer Codes
Source: Sci Rep. 2017 Sep 8;7:11003. doi: 10.1038/s41598-017-11266-1 (PMC5591216; doi:10.1038/s41598-017-11266-1)
Supplement: Supplementary file 1 — Supplementary Material [file 41598_2017_11266_MOESM1_ESM.pdf]

Supplementary Materials to "Deep Neural Network Probabilistic Decoder for Stabilizer Codes"

Stefan Krastanov
Department of Physics, Yale University
Yale Quantum Institute

Liang Jiang
Department of Applied Physics, Yale University
Yale Quantum Institute

Performance of sampling through "hard-decision message passing"

In the main text we mention that sampling from the neural network can be done in two different manners: either through naive resampling in which the entire "candidate error vector" sample is scraped if it does not reproduce the measured syndrome or through a more advanced "hard-decision message passing" which provides for a significant speedup. The message passing works by checking which qubits belong to the violated syndrome components (i.e. which variable nodes are connected to the active check nodes on the Tanner graph) - in the resampling step, only those qubits are resampled, hence preserving the already properly decoded components of the error vector. The following figure, similar to the figures in the main text, demonstrates the speedup for a resampling performed on a deep network decoding the 5x5 code. In order to also compare the hard-decision message sampling on its own, without the use of a neural network (i.e. the way message passing is currently used in classical LDPC decoders), we also show a curve where the message passing sampler is run on an untrained network (as expected, it performs poorly - it is known that message passing on its own does not work well for quantum LDPC codes due to the presence of 4-cycles in the Tanner graph).

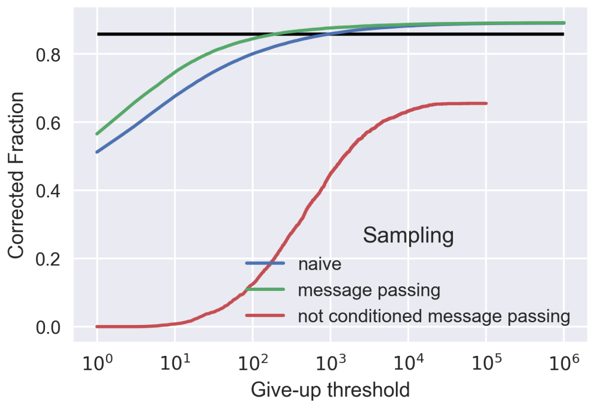

Performance vs depth of the neural network

The following figure, similar in style to figures in the main text, shows the growth in performance as we increase the depth of the neural network (the example is for a 5x5 toric code). As discussed, a point of diminishing returns is reached, where the expense of adding more layers outweighs the minor gains in performance.

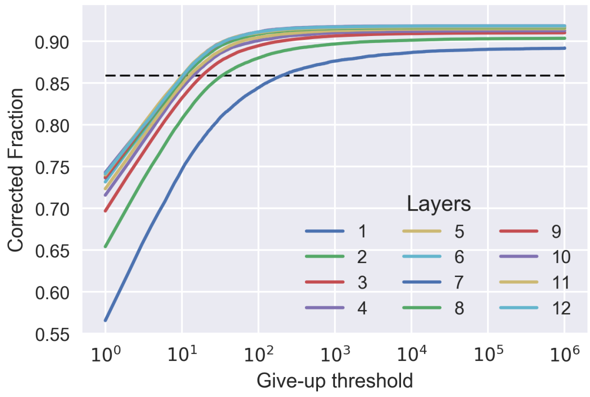

Software package

The software described in the main text is available from the authors. It is based on the Keras and Tensorflow NN libraries and can run on GPU accelerators. The software provides independent command line utilities that can be used to design and evaluate deep neural network decoders with arbitrary hyperparameters. Upon request we would be happy to provide pretrained networks for any reasonably sized code of interest.
